# Understanding Compliant Behavior During a Pandemic: Contribution From the Perspective of Schema-Based Psychotherapy

**DOI:** 10.3389/fpsyg.2022.805987

**Published:** 2022-02-07

**Authors:** Chino José Offurum, Max Leibetseder, Brigitte Jenull

**Affiliations:** ^1^Faculty of Psychotherapy Science, Sigmund Freud University, Vienna, Austria; ^2^Institut für Verhaltenstherapie [Cognitive Behavior Therapy Training Institute] – AVM, Salzburg, Austria; ^3^Department of Clinical Psychology, University of Innsbruck, Innsbruck, Austria; ^4^Department of Psychology, University of Klagenfurt, Klagenfurt, Austria

**Keywords:** compliance, coping, core psychological needs, COVID-19 pandemic, schema-based psychotherapy

## Abstract

**Objective:**

The current study examined whether compliance with anti-pandemic measures during the COVID-19 pandemic relates to (a) importance of the fulfillment of core psychological needs, namely, *relationship, self-esteem, efficacy*, and *pleasure*; (b) coping behavior styles, namely, *surrender, self-soothing, divert attention*, and *confrontation*; and (c) worries or concerns beyond COVID-19 which may impair wellbeing.

**Methods:**

This study used a cross-sectional design and online survey data from responses to a structured questionnaire developed within the theoretical framework of schema-based psychotherapy on psychological needs and coping behavior styles from 740 participants in Central Europe and West Africa.

**Results:**

Analysis indicated that people with the psychological needs of “pleasure” and “efficacy” and the coping style of “surrender” were more likely to comply with anti-pandemic measures. We also found that people with the coping style of “confrontation” were less likely to comply. There were no statistically significant relationships between compliance and “relationship,” “self-esteem,” “self-soothing,” “divert attention,” and “existential concerns.”

**Discussion:**

Our findings indicate that how likely a given individual is to comply with prescribed pandemic countermeasures varies based on their specific psychological needs and behavior styles. Therefore, to control contagion during a pandemic, authorities must recognize the relevance of human need fulfillment and their behavior styles and accordingly highlight and encourage admissible and feasible actions. The findings demonstrate that some individual differences in core psychological needs and coping behavior patterns predict compliance behavior.

## Introduction

The COVID-19 pandemic has become an unprecedented global threat. The Emergency Committee of the World Health Organization (2020a) declared it a public health emergency of international concern on January 30, 2020, and a pandemic on March 11, 2020. The virus that causes COVID-19, scientifically named SARS-CoV-2, is highly contagious, and the risk of transmission particularly depends on individual and collective behavior. While the disease may be mild for most patients, the risk of hospitalization and mortality increases with age and some underlying conditions (Robert Koch Institut, 2021); the surge of infections and congestion of intensive care units by severe cases have encumbered healthcare infrastructure worldwide. To combat the pandemic, the World Health Organization (2020b) and the disease control organizations of national governments have recommended a series of precautionary measures, including restrictions on travel, public gatherings, in-school teaching, and face-to-face interactions. Several of these measures were unusual prior to pandemic and are fiercely debated both in public and private as they have raised important questions about the meaning of life when basic needs are not met; the intrusive nature of preventive policies with their potential psychological and social consequences; and the risk of creating a new normal with restricted human rights and liberties, among other controversies.

In this study, we sought to understand *compliant* behavior during the pandemic in the light of schema-based psychotherapy with regards to (a) the fulfillment of core psychological needs (CPNs) and (b) coping behavior styles (CBSs). Both concepts are pillars of schema-based psychotherapy. Further, we sought to understand these in the context of people’s concerns beyond the pandemic, as potential impediments to wellbeing, analog to the context of concepts of schema-based therapy. As the pandemic may not be the only present threat to wellbeing, we viewed it reasonable to investigate the effects of diverse concerns people have on compliance beyond the pandemic.

This study was motivated by the controversies mentioned above and the fact that pandemics may occur more frequently in the future (*cf*. United Nations, 2020). On the one hand, the anti-pandemic directives tend to facilitate survival, and humans by nature strive to survive. On the other hand, some people comply while others do not. Hence, we are furthermore motivated to understand the reason. From a therapeutic point of view, we infer that compliance can also have a negative connotation when clients should thereby be constrained to grievously suppress their own needs, which can lead to clinical disorders, psychological distress, or impairment of wellbeing. In addition, although there have been several studies on individual coping strategies during the pandemic, to the best of our knowledge, there is no study aimed at understanding compliant behavior during a pandemic from the perspective of schema-based psychotherapy.

## Theoretical Framework

Schema-based psychotherapy (also known as schema therapy) is becoming increasingly popular both among psychotherapy researchers and practitioners. There are two independently developed influential traditions. One tradition particularly conceptualized as *consistency-schema theory* is given by Grawe (2000, 2004). The other tradition, conceptualized as *schema-mode-model*, is given by Young et al. (2007).

### Core Psychological Needs

According to the consistency theory (Grawe, 2000; *cf*. Fries and Grawe, 2006; *cf*. Grosse-Holtforth et al., 2008), the striving for consistency of psychic processes is a superordinate principle of psychic functioning. Among others, the core teachings of the consistency theory model are that humans strive for the equilibrium of gratification of the basic psychological needs and that incongruence (a significant form of inconsistency) is a major cause of the development and maintenance of psychopathological symptoms and poor wellbeing. In this theory, Grawe developed the concept of *motivational schemata*, where he differentiates between “avoidance motivational goals” (which are defined as mental representations of undesired transactions with the environment) as opposed to “approach motivational goals” (which are representations of desired transactions). The function of *approach motivational goals* is to ensure that basic needs are satisfied, while *avoidance motivational goals* serve to protect the individual from repetition of aversive experiences. However, if the avoidance schema dominates an individual’s life, what originally had the function of protecting the individual’s needs (e.g., being separated from others and being criticized) can paradoxically hinder the satisfaction of these same needs. In general, the schemas, which have neurological imprinting, are viewed by Grawe as organized units of psychological regulation for the purpose of reduction of complexity through classification in patterns, according to which they thus govern behavior. In particular, a person’s *plan structur*e includes all the conscious and unconscious strategies developed throughout life to instrumentally fulfill one’s needs. Thus, in *vertical analysis* (generally in behavioral therapy) or *plan analysis* (particularly in the consistency-schema theory) gratification of basic needs is at the topmost level; accordingly, these needs are the ultimate driving factors of human behavior (*cf*. Caspar et al., 2005; Caspar, 2009, 2018).

The doctrine of basic psychological needs teaches that certain requirements must be fulfilled to sustain a psychological healthy life beyond mere physical existence (Becker, 1995). In his consistency theory, Grawe (2004) proposed the importance of balance in the fulfillment of CPNs, which he regards as the highest *desired value* (“*Sollwert*”) of psychological activity. He describes these basic psychological needs as the need for attachment, increasing self-esteem, orientation and control, and gaining pleasure and avoiding displeasure. He views them as pervasive, in that they permeate all mental events.

The innate *desired value* of the need described in psychology as bonding, connection, or connectedness is stated as the basic need for “*relationship*.” Grawe’s (2004) need for “*self-esteem*” is often misunderstood as the need for *permanently elevating* one’s self-worth (“*Selbstwerterhöhung*”). However, it refers to the innate need for an *elevated* self-value (Offurum, 2019). This CPN comprises self-esteem, dignity, respect, autonomy, and self-determination. The need for orientation and control should be broadly understood as the innate *desired value* of the need to have “freedom of action,” self-efficacy, and locus of control or actionability, and includes the need for performance and achievement (Offurum, 2019, 2021). This need is referred to as “*efficacy*,” “handling,” or “actionability” in the current study. The basic need for “*pleasure*” includes the need for enjoyment, pleasurable experiences, play, fun, relaxation, ease, and esthetics. Grawe emphasizes that the underlying concepts, not the names, are decisive; thus, diversion in terms has existed and may still exist in psychology. To our knowledge, there is no questionnaire in schema-based therapy to examine basic psychological needs during a pandemic. Our items for this study were thus formulated based on preliminary interviews in this field (Offurum, 2021).

### Coping Behavior Styles

Young’s schema approach (Young et al., 2007; *cf*. Lobbestael et al., 2007; *cf*. Roediger, 2011) is based, among other concepts, on the concept of the *early maladaptive schemas (EMS)*, *maladaptive coping styles and responses*, and the *mode model*. The early maladaptive schemas are emotionally anchored unconscious maladaptive self-defeating core cognitive patterns that an individual develops during childhood, which are elaborated throughout one’s lifetime. Young has presented 18 EMSs like *abandonment*, *mistrust/abuse*, *enmeshment*, *grandiosity*, *hypercriticalness*, and *emotional deprivation* (*neglect*). One could view these as person’s *sore spots*, which seem compatible with the *avoidance schemas* in Grawe’s concept. In Young’s theory, behavior is embedded in the three coping styles that form the second main feature in his model. Therein, individuals develop dysfunctional coping styles in order to cope with challenges when their schemas are triggered. These styles are maladaptive in his concept because, although initially they were strategies in coping with painful experience in childhood, paradoxically they are later applied at inadequate situations, thereby contributing to reinforcement and perpetuation of the maladaptive schemas.

In psychology, the transactional model of coping with psychological stress is well established (Folkman et al., 1986). If coping patterns are applied across situations and maintained over a long time, situational (reaction) *states* can transcend time and situations to become personality *traits*. Therefore, coping can be seen as both a situational and a trans-situational response to challenges. It can be studied from different perspectives, such as personality disposition (habitual pattern or schema), situational ego-state (mode), or systemic (transactional dynamic; *cf*. Rexrode et al., 2008).

In psychotherapy, current conceptualizations of coping correspond to the theory of *ad hoc coping strategies* (Horney, 1992) in psychoanalysis, according to which humans have three *ad hoc* strategies to cope with the world at their disposal. The first is the strategy of *moving toward* people. Here, the person exhibits consent or approval and considers others but neglects themself (Smith, 2007). In systemic psychotherapy, Satir (1988) calls this the placating communication style. The current study uses the term *subjugation*. Horney’s second strategy, *moving against* others, emphasizes hostility and aggression. Here, life is considered a struggle and the individual exhibits the coping strategy of *fighting*, corresponding to Satir’s (1988) blaming communication style. The individual primarily considers themself while neglecting others in the third strategy, *moving away*, the individual *flees*, separating themself and potentially becoming neurotically detached from others, and preventing anyone or anything from touching or mattering to them (Smith, 2007). Horney does not view these *ad hoc* strategies as invariably maladaptive.

Schema therapy, as influenced by Young et al. (2007), emphasizes the maladaptive nature of coping patterns and contains three methods for adapting to one’s schema. The first strategy is known as “*surrender*” (in German, the term *Erduldung*, or “*endurance*,*”* is preferred; see Roediger, 2011). The second style is termed “*confrontation*” *or* “*overcompensation*,” and the third is called “*avoidance*.” A bifocal approach is useful to examine these maladaptive coping behavior styles. The primary perspective defines them as behavioral strategies *vis-à-vis* the overwhelming *feeling* of psychological distress by maladaptive schemas. In the secondary perspective, however, they are also used to explain behavioral maneuvers *vis-à-vis* the *counterparts* that trigger the maladaptive schemas. Regardless of perspectives, the styles are regarded as behavioral responses to the schemas from which they differ. Further, while “surrender” (“subjugation” or “placating”) and “confrontation (“fight” or “counter attack”) exhibit proximity, avoidance can be seen in a passive and active manner: active in the sense of fleeing, or diverting attention (rationalizer or distractor style), and passive in the sense of *pacifying* or *self-soothing*. The categorization may seem ambiguous, as some authors (e.g., Atkinson, 2012; Faßbinder et al., 2016) write that *freezing* belongs to the same category as *surrender/subjugation*, while others (Roediger and Zarbock, 2015) categorize it under *avoidance*.

Existing questionnaires explore coping behaviors from various theoretical perspectives. The Ways of Coping Questionnaire is based on Lazarus’s cognitive theory of psychological stress and coping (Folkman et al., 1986). Similar to various adaptations (Sawang et al., 2010; Senol-Durak et al., 2011; Kolokotroni, 2014), the items used in our study are based on Folkman et al.’s (1986) concept, which have been incorporated within the framework of *schema-oriented psychotherapy*.

### Worry or Concerns

Both Grawe’s and Young’s traditions of schema-based psychotherapy dwell in the context of impairment of wellbeing, here conceptualized as worry or concerns in a non-clinical context. Concern or worry may be attributed to cognitive-emotive preoccupation with uncertainty, anxiety, and apprehension about the future. Excessive and uncontrollable worry constitutes the main diagnostic criterion for generalized anxiety disorder. Pathological worry is experienced as emotionally distressing and impairing. Although uncomfortable and potentially detrimental to health, worry can have the advantage of helping people avoid or solve problems (Borkovec et al., 1983). With novel threats, as in the present pandemic, and when individuals do not feel in control of the risk, they are more concerned (Carlucci et al., 2020). The current study focuses on the concept of worry or concern, regardless of pathological status. We investigated whether concerns/worry about issues beyond the pandemic affected compliance with pandemic-related restrictions by asking participants how worried they were about the following: (a) health issues, (b) crime and social insecurity, (c) setback at school or work, and (d) financial or economic problems.

### Compliance

Compliance is the dependent variable in this study. In medical practice, compliance or adherence describes the degree to which a patient follows medical advice. In this research, compliance refers to the application of therapeutic suggestions, both for treatment and prevention. Compliance with pandemic-related recommendations can generally be compared with adherence to medical guidelines. However, while non-adherence to personal medical advice may have no legal repercussions, defiance of pandemic regulations may carry severe legal consequences because the success of these measures rests on the individual’s compliance.

Although governmental responses to the pandemic have varied, most are comparable in their severity, duration, and types (Ritchie et al., 2021). To investigate compliant behavior, we employed the World Health Organization’s (2020b) recommendations, representing the cardinal guidelines imposed worldwide: regularly and thoroughly washing one’s hands with soap and disinfecting them to eliminate germs and viruses; wearing a mask or face shield in public; avoiding close contact, particularly shaking hands and hugging; cleaning and disinfecting surfaces frequently, especially those which are regularly touched, such as door handles, faucets, and phone screens; reduction of public transport; avoiding crowds; and fostering one’s immunity. At this stage of the pandemic, no vaccine was available, and because eradication of the virus is not possible, the main purpose of these prescriptions was to “flatten the curve” of its spread to prevent the congestion of intensive care units and prevent triage.

There have been many studies on compliance during the present pandemic: Some studies (Brouard et al., 2020; Carlucci et al., 2020; Raude et al., 2020) discuss the socio-demographics of individuals with regard to their compliance. Others, like Blais et al. (2021) and Dinić and Bodroža (2021), have studied personality differences that may relate to compliance. Farias and Pilati (2021) studied political ideology, while some others (Plohl and Musil, 2021; Wright et al., 2021) studied trust in science, government, and/or medical professionals. Baloran (2020) and Wang et al. (2020) studied coping during this pandemic. Orgilés et al. (2021) and Donato et al. (2020) studied disturbance, worry, or concern. Eisenbeck et al. (2021) studied meaning-centered coping. While these studies are very informative, none examine behavior in the light of an (influential) psychological/psychotherapy concept like schema-based therapy; thus, the present study aims to fill this gap.

### Hypotheses

This study sheds light on the effect of CPNs (Hypotheses 1–4), coping styles (Hypotheses 5–8), and concerns other than the pandemic (Hypothesis 9) on participant’s compliance with anti-pandemic measures (Supplementary Figure 1).

*Hypotheses* 1, 2: The more essential the fulfillment of fundamental needs of (i) (“relationship” or “self-esteem”) are during the pandemic, the less compliant people are as: H0[1, 2]: βi ≥ 0 versus H1[1, 2]: βi < 0.

*Hypotheses* 3, 4: The more essential the fulfillment of fundamental needs of (i) (“efficacy” or “pleasure”) are during the pandemic, the more compliant people are as: H0[3, 4]: βi ≤ 0 versus H1[3, 4]: βi > 0.

*Hypotheses* 5, 6: The higher the value of the coping behavior style of (i) (“self-soothing” or “surrender”) are, the more compliant people are as: H0[5, 6]: βi ≤ 0 versus H1[5, 6]: βi > 0.

*Hypotheses* 7, 8: The higher the value of the coping behavior style of (i) (“confrontation” or “divert attention”) are, the less compliant people are as: H0[7, 8]: βi ≥ 0 versus H1[7, 8]: βi < 0.

*Hypothesis* 9: With regard to concerns, we hypothesize that the stronger the concerns (i) are, the more compliant people are as: H0[9]: βi ≤ 0 versus H1[9]: βi > 0.

βi = Regression coefficient of individual predictor.

## Materials and Methods

### Study Design

Having developed the items for our study with care and in line with the theoretical framework mentioned above, we performed “face-to-face item-pretests.” These were performed *via* video telephony due to movement and travel restrictions. The interviewers were similar to the intended participants, being males or females who were not experts in the field of research. We chose five individuals from each area targeted for dissemination and provided them with the pretest questions. While completing the questionnaire, they were repeatedly requested to be very critical and to comment on anything that crossed their mind (simply think aloud approach); especially, where something seemed unclear or ambiguous. Notes of the testers’ comments were taken, and further questions asked (verbal probing approach) to ensure, for example, that the questions were understood and answered in terms of the construct. The questionnaire was improved accordingly after each pretest followed by further rounds of pretests.

This method of pretesting as provided by the applied software (SoSciSurvey, 2020) corresponds to the concept of cognitive interviews. In cognitive interviews, ensuring validity of the research instrument involves examining how respondents (a) understand the question, (b) retrieve relevant information, (c) judge their answer, and (d) assign their response into the questionnaire (Ryan et al., 2012). The goal was therefore to utilize the information during the various pretests to improve the quality of the questionnaire, and thus, the quality of responses. Cognitive interviews were primarily developed to test each question in a questionnaire but not to check the technical functionality of a questionnaire. For this reason, it was supplemented with further “online pretests” (pretest without the researcher’s presence). The pretest hyperlink was thus distributed to testers who accessed the questionnaire without the involvement of the researchers. They were requested to leave comments about the questionnaire in the test-comment area provided by the software. The questionnaire was improved accordingly and as a result, the best qualified items were selected. Finally, additional tests of technical functionalities were performed using a PC, tablet, and smart phone with various browsers, prior to questionnaire administration.

In September 2020, we launched the comprehensive cross-sectional online survey. The questionnaire was provided on the survey platform *SosciSurvey* and, for security and data protection reasons, hosted on the server of the Sigmund Freud University, Vienna, Austria. The survey was conducted until January 2021.

### Participants

The prospective participant had to be a literate individual aged 18 years or above who had a PC or smartphone and Internet access at the time. Individuals under 18 years were explicitly excluded, while those who were not literate or lacked Internet access or access to a PC or smartphone were *de facto* excluded. Our objective was to recruit participants from Central Europe, West Africa, and America, along with the help of our research colleagues in those regions. However, due to unforeseen circumstances, the colleague in America was unable to disseminate the questionnaires. In the other locations, the the invitation with link to participate was disseminated, particularly *via* the administration offices of educational institutions, to all members of the institution (not just students). We additionally disseminated directly to our students or clients during lectures. The invitation contained a request to forward the link to friends or colleagues who met the inclusion criteria. The research was conducted in accordance with the Code of Ethics of the World Medical Association (Declaration of Helsinki, 1964) and with the approval of the Ethics Commission of the Institut *für Verhaltenstherapie* (Institute for Cognitive Behavior Psychotherapy Training and Research), AVM, Salzburg, Austria. Participants were informed of the study’s purpose and procedure, guarantee of anonymity and data protection, and voluntariness of participation, and informed consent was obtained from all respondents prior to participation. To answer our research questions, we extracted the relevant sections from the comprehensive survey. The questionnaire was structured such that no missing items were allowed. After data cleaning (Leiner, 2019), 740 responses were analyzed.

Respondents’ demographics were as follows: 450 women (60.8%) and 290 men (39.2%); 380 from Austria (51.4%), 44 from Germany (5.9%), 290 from Nigeria (39.2%), and 26 from other countries (3.5%); mean age was 32.06 years, median of 28.50, standard deviation of 12.547, variance of 157.429, minimum of 18, and maximum of 83 years.

Regarding the COVID-19 status in the countries where major participants were situated, Nigeria and Austria were in their second wave of infections, and Germany was in its third (or extended second) wave. Nigeria restricted public gatherings between 10 and 100 people and workplace closures were recommended. In Austria and Germany, public gatherings were limited to less than 10 people and workplace closures were required, except for essential workers. In all three countries, school closures were mandatory, and face covering policies were implemented in all public spaces. According to a stringency index where 100% denotes the most strict protocols, Nigeria, Austria, and Germany scored 58.33, 82.41, and 82.41%, respectively, at the time. The nine metrics used to calculate the Stringency Index are school closures, workplace closures, cancelation of public events, restrictions on public gatherings, closures of public transport, stay-at-home requirements, public information campaigns, restrictions on internal movements, and international travel controls (Ritchie et al., 2021).

Based on age, social-generational groups were represented as follows: 254 participants (34.3%) belonged to Generation Z, with a maximum age of 23 years; 294 (39.7%) to Generation Y (millennials), aged 24–39; 151 (20.4%) to Generation X at ages 40–55; 41 (5.5%) to the Baby Boomers; and traditionalist/silent generation, aged 56–92.

Thirty-five (4.7%) participants had junior secondary education (e.g., GED and GCSE) or lower, 249 (33.6%) had senior secondary education or a high school diploma/A-levels, 121 (16.4%) had an undergraduate diploma degree (OND, HND, or equivalent), 175 (23.6%) had a bachelor’s degree (BA, BSc, or equivalent), 104 (14.1%) had a master’s degree (MA, MSc., MPhil., or equivalent), 23 (3.1%) had a doctorate (PhD or equivalent), and 33 (4.5%) had other education or qualifications (Supplementary Table 1).

### Instrument, Procedure, and Preliminary Data Analyses

The Statistical Package for Social Science was employed for data analyses. To elicit meaningful and valid meta-scales based on the items, and to empirically verify our hypotheses, we conducted exploratory factor analyses (EFAs) of (1) compliance with the anti-pandemic recommendations, (2) the CPNs, (3) the CBSs, and (4) concerns. The EFAs were conducted to establish unidimensional scales, ensuring that an individual scale is not influenced by confounding factors, thereby obtaining valid measurements of the underlying concepts.

These preliminary analyses were performed according to the following unitary pattern: (a) presenting the items and checking the suitability of the data as per Bartlett’s and Kaiser–Meyer–Olkin (KMO) tests; (b) factor analyses using the scree plot and rotated component matrix to determine the optimal number of factors, followed by the interpretations; and (c) defining suitable terms for each factor.

#### EFA of Compliance

We applied the World Health Organization (2020b) pandemic prevention guidelines at the time to investigate compliant behavior. On a five-point Likert scale (1 = never; 5 = always), we rated participants’ compliance with the following items: “I cleanse my hands with soap and water and/or use hand-sanitizer more regularly,” “I wear a mask or face shield at public premises to protect myself and/or others,” “I avoid shaking hands or hugging people,” “I clean or disinfect surfaces I might touch more often,” “I have stopped or reduced traveling by public transport,” “I avoid group events or crowded places,” and “I am trying to boost my immunity (e.g., with vitamins, healthy food, sports)” (Supplementary Table 2). To check the suitability of the data for the EFA, the KMO criterion and Bartlett’s test of sphericity were calculated. Bartlett’s test yielded *p* ≤ 0.001, indicating high significance. The KMO value was 0.84, ensuring highly suitability for the EFA.

To verify the unidimensionality of compliance items, a scree plot analysis was conducted (Supplementary Figure 2). The scree plot showed that only one eigenvalue exceeds the Kaiser criterion of 1, thus confirming the unidimensionality of the compliance items being suitable to be combined to one scale. The unidimensional factor is termed “compliance.”

#### EFA of Relevance of Fulfillment of CPNs

On a five-point Likert scale (1 = not; 5 = very), participants rated the necessity for them to get their CPNs fulfilled during/despite the pandemic. The items used in this analysis are listed in Supplementary Table 3. To check the suitability of the data for the EFA, the KMO criterion and Bartlett’s test of sphericity were employed. Bartlett’s test yielded *p* ≤ 0.001, indicating high significance. The KMO value was 0.90, ensuring high suitability for the EFA.

To elicit the optimal number of factors, a scree plot was drawn, and the Kaiser criterion was applied (Supplementary Figure 3). Based on the scree plot and Kaiser criterion, an eigenvalue of the factor above 1 indicated four to be the optimal number of factors. Thus, EFA was conducted using four factors. Further, to provide an intuitive interpretation of the analysis and the best possible separation among the four factors, a varimax rotation was applied.

The EFA results (Supplementary Table 3) show that our items can be meaningfully grouped following the fundamental theory of CPNs in schema-oriented psychotherapy. These factors are named “efficacy,” “pleasure,” “relationship,” and “self-esteem.” Our empirical analysis thus supports the underlying structures of CPNs theorized above. In contrast to expectations, the items BV04_10, BV05_04, BV05_05, BV05_08, and BV05_10 did not follow the structure of previously theorized scales. To analyze the validity of the categorization of these items, different rotation and extraction methods were applied. Three (BV05_04, BV05_08, and BV05_10) of the items were unable to meet both theoretically and statistically meaningful groupings to justify their inclusion and were, therefore, dropped. Regarding the variance explained by the factors, each factor explains approximately the same amount, indicating a similar level of importance.

#### EFA of CBSs

On a five-point Likert scale (1 = never; 5 = always), participants rated how they dealt with the challenges of the present pandemic. The items used in this analysis are listed in Supplementary Table 4. With respect to coping based on behavioral style, Bartlett’s test yielded *p* < 0.001, indicating high significance. The KMO value was 0.85, ensuring the suitability of the data for conducting an EFA.

To elicit the optimal number of factors, a scree plot was drawn, and the Kaiser criterion was applied (Supplementary Figure 4). Based on the scree plot and Kaiser criterion, an eigenvalue of the factor above 1 indicated four to be the optimal number of factors. Thus, EFA was also conducted using four factors. To provide an intuitive interpretation of the analysis and the best possible separation among the four factors, a varimax rotation was applied.

As shown by the results (Supplementary Table 4), the items can be meaningfully grouped and the factors established can be identified with the terms “self-soothing,” “confrontation,” “surrender,” and “divert attention.” However, two items (BV06_07 reflecting “escape” and BV07_09 reflecting “confrontation”) were theoretically unsuitable to justify their inclusion in the group “self-soothing” and were thus dropped.

#### EFA of Concerns

In this analysis, the participants’ concerns for health issues, crime and social insecurity, setback at school or work, and financial or economic problems rated on a five-point Likert scale (1 = not at all; 5 = very much) were evaluated. Bartlett’s test yielded *p* < 0.001, indicating high significance. The KMO value was 0.705, ensuring the suitability of the data for conducting an EFA. The scree plot analysis (Supplementary Figure 5) showed the unidimensionality of concern items, thus suitable to be combined to one scale. The unidimensional factor is termed “existential concerns.”

## Results

### Overview of the Scales

As shown in Table 1, descriptive parameters, such as means, standard deviations, skewness, and kurtosis of the scales, do not exhibit any irregularities. Generally, Cronbach values of approximately 0.65 are considered moderate but acceptable, mainly where small item-numbers are involved in exploratory research (Hinton et al., 2004; Hair et al., 2014). With values ranging from 0.65 to 0.83, the Cronbach’s alphas of our scales, therefore, indicate acceptable to very good reliability. The corresponding Omega values are presented in the table. The table also shows that all significant intercorrelations have positive intercorrelations, as theoretically expected. Thus, good criterion validity is assumed.

**Table 1:** Intercorrelations of scales based on exploratory factor analysis.

Descriptive parameters. Reliabilities and intercorrelations of scales																	
S. No.	Scale	*M*	*SE*	*SD*	Skewness	Kurtosis	*Ω*	1	2	3	4	5	6	7	8	9	10
1.	Compliance	3.76	0.03	0.81	−0.52	−0.21	0.83	(0.83)									
2.	CPN-relationship	3.43	0.03	0.81	−0.15	−0.33	0.75	0.275^***^	(0.74)								
3.	CPN-self-esteem	3.14	0.03	0.89	−0.06	−0.52	0.67	0.124^***^	0.223^***^	(0.66)							
4.	CPN-efficacy	3.65	0.03	0.76	−0.33	−0.05	0.73	0.242^***^	0.423^***^	0.498^***^	(0.73)						
5.	CPN-pleasure	3.61	0.03	0.76	−0.28	−0.05	0.78	0.326^***^	0.459^***^	0.353^***^	0.563^***^	(0.78)					
6.	CBS-self-soothing	2.32	0.03	0.91	0.62	−0.08	0.73	0.093^*^	0.244^***^	0.339^***^	0.089^*^	0.091^*^	(0.73)				
7.	CBS-confrontation	3.00	0.03	0.87	0.20	−0.21	0.68	−0.036	0.032	0.493^***^	0.364^***^	0.249^***^	0.371^***^	(0.68)			
8.	CBS-surrender	2.93	0.03	0.82	0.18	−0.06	0.67	0.314^***^	0.189^***^	0.298^***^	0.194^***^	0.208^***^	0.422^***^	0.317^***^	(0.65)		
9.	CBS-divert attention	3.31	0.03	0.92	−0.26	−0.07	–	0.237^***^	0.317^***^	0.423^***^	0.384^***^	0.469^***^	0.353^***^	0.358^***^	0.375^***^	(0.69)	
10.	Existential concerns	3.22	0.04	1.06	−0.10	−0.90	0.79	0.004	0.157^***^	0.405^***^	0.248^***^	0.185^***^	0.274^***^	0.333^***^	0.211^***^	0.210	(0.77)

* *p* ≤ 0.05 and

*** *p* ≤ 0.001.

*N = 740; Diagonal: Cronbach’s alpha; CPN, core psychological need; and CBS, coping behavior style, existential concerns. Omega (Ω) using macros by Hayes on SPSS. Omega for “divert attention” not calculable due to unavailability of minimum number of items*.

### Multiple Linear Regression Suitability Analysis

Multiple linear regression is a statistical method to estimate the relationship between several explanatory (independent) variables and one observed (dependent) variable. To provide valid results, the linear multiple regression is based on several statistical assumptions, such as linearity of the associations (multivariate), normality (data is symmetrically distributed with no skew), no multicollinearity, and homogeneity of variance (homoscedasticity of residuals).

Linearity of the association was double-checked by partial added plots not indicating any better association than the (applied) linear one. The assumption of normally distributed residuals was visualized by a P–P plot indicating no violation of the assumption. Possible multicollinearity problems were double-checked by calculating the variance inflation factor (VIF). With VIF values of <2.81, no multicollinearity problems were identified. Minor heteroscedasticity issue was detected with the scatterplot between fitted and actual values. Accordingly, heteroscedasticity consistent standard error (Hayes and Cai, 2007) was applied to improve the model and to ensure the results’ validity. Consequent to these preliminary analyses, the model’s result can be presumed to be valid. Finally, covariates, such as age, gender, education, savings, and country, were included to control for socio-demographic variables.

### Test of Hypotheses: Effect of CPNs, CBSs, and Concerns on Compliance

As shown in Table 2, the overall regression model achieved an *R*^2^ of 0.232, with a significant value of *p <* 0.001, indicating the relevant effects measured within the model.

**Table 2:** Regression analysis of the effect of CPNs, CBSs, and concerns on compliance.

Dependent variable: Scale compliance			*R*^2^ = 0.232	*F*_(16;684)_ = 12.640	*p* > |F| = 0.000^***^
Items	*Coeff*.	*SE* (*HC*)	*Coeff* (*Std*.)	*t*	*p > |t|*
(Constant)	1.923	0.248		7.741	0.000^***^
Scale CPN-relationship	0.053	0.046	0.053	1.158	0.247
Scale CPN-self-esteem	0.032	0.045	0.034	0.704	0.482
Scale CPN-efficacy	0.116	0.058	0.107	1.988	0.047^*^
Scale CPN-pleasure	0.193	0.051	0.18	3.824	0.000^***^
Scale CBS-soother	0.024	0.042	0.027	0.562	0.574
Scale CBS-confrontation	−0.165	0.047	−0.177	−3.531	0.000^***^
Scale CBS-surrender	0.31	0.042	0.309	7.393	0.000^***^
Scale CBS-divert attention	0.037	0.037	0.042	1.006	0.315
Scale CBS-existential concerns	−0.047	0.034	−0.061	−1.377	0.169

* *p* ≤ 0.05 and

*** *p* ≤ 0.001.

*HC, Heteroscedasticity consistent standard errors; Covariates: Age, gender, educational level, country, and savings; CPN, Core psychological need; and CBS, Coping behavior style*.

With a significant value of *p* of 0.047 and a regression coefficient of 0.116, a positive effect of psychological need for “efficacy” was confirmed. Thus, the more essential that the basic psychological need is to handle (self-efficacy), the higher compliance can be expected.

With a significant value of *p* < 0.001 and a regression coefficient of 0.193, the positive effect of psychological need for “pleasure” was verified. Thus, the more essential the fulfillment of the CPN for “pleasure” is during the pandemic, the higher the expected compliance with policies.

With a significant value of *p* < 0.001 and a regression coefficient of −0.165, the negative effect of “confrontation” was verified. Thus, the higher the coping behavior style of confrontation during the pandemic, the lower the expected compliance with policies.

With a significant value of *p* < 0.001 and a regression coefficient of 0.310, a positive effect of “surrender” was confirmed. Thus, the higher be coping behavior style of surrendering in dealing with the pandemic, the higher compliance can be expected.

With respect to the standardized coefficient, the effect size of “CBS-surrender” on “compliance” (0.309) was greater than that of “CPN-pleasure” on “compliance” (0.180), followed by “CBS-confrontation” (−0.177) and “CPN-efficacy” (0.107). In the case of other effects, such as “relationship,” “self-esteem,” “self-soothing,” “divert attention,” or “existential concerns,” no significant effect on compliance was confirmed.

## Discussion

### Core Psychological Needs

This study aims to contribute to understanding behavior during the pandemic in the light of theories of schema-based therapy. Grawe’s tradition teaches that topmost motivational factor of human behavior lies in the gratification of the basic needs, as demonstrated in plan analysis (Caspar, 2009, 2018). Accordingly, the individual’s motivation to comply with measures during the pandemic would depend on their topmost “plan,” and the topmost level of a person’s plan structure consists of the psychological needs essential to them at the given time. These needs can be categorized into the core needs of (a) “relationship,” (b) “self-esteem/dignity/recognition/self-determination,” (c) “efficacy/handling/actionability,” and (d) “pleasure/easiness/gaudium” (Grawe, 2004; Offurum, 2019).

We therefore assessed the importance of the core psychological needs during the pandemic and tested the hypotheses that with the topmost motivation being the gratification of the CPN for “relationship” or “self-esteem,” individuals would not comply with the anti-pandemic policies, but with the topmost motivation being “efficacy” or “pleasure,” individuals would indeed comply with the anti-pandemic policies.

These assumptions were based on the premises that, due to the nature of the pandemic (contagion through contact), the measures restrict contact, which is a core aspect of relationship and that, due to the nature of sanctioning, individuals whose topmost plan during the pandemic was self-esteem (dignity) were highly challenged. However, for those whose topmost plan toward the pandemic lies in the category of efficacy (actionability), the measures provide an opportunity to act; and individuals whose topmost plan during pandemic was to experience pleasure and avoid pain would comply in order to avoid the pain of the viral infection.

Our results show that the four-factor categorization of basic needs according to the conceptualization of Grawe’s tradition of schema-based therapy is adoptable. Further, our results show that the higher the importance of fulfillment of the CPN for *efficacy*, the higher compliance can be expected. This seems to demonstrate the driving factor of efficacy (actionability, control, or achievement of solution) amidst the threats of the pandemic. Perhaps, those whose topmost plan toward the pandemic lies in efficacy cannot endure being passive toward the threats.

Our results also show the higher the importance of fulfillment of the CPN for *pleasure*, the higher compliance can be expected. This seems to suggest that restrictions on festivities must not have prevented individuals from having pleasure, or that avoiding pain, which is a core aspect of the CPN for pleasure, must be a driving force for compliance. However, we found no statistically significant relationship between the CPNs of relationship and self-esteem with compliance. Concerning relationship, this may be because the restrictions of human contact to curtail the pandemic may have particularly jeopardized the fulfillment of needs for relationship, but that relationship *via* digital technology must have partially compensated the deficit, however not enough for statistically significant positive effect on compliance. Concerning self-esteem, this may be because for these people, maintaining elevated self-esteem was indeed essential at the time, but unlike the factor “relationship,” self-esteem may not have been very much challenged by these restrictions, particularly not by those on human contact. However, the self-esteem of people must have still not been considered enough by authorities to provide for a statistically significant positive effect on compliance.

### Behavior Styles

Young’s tradition of schema-based therapy differentiates between the maladaptive *coping styles and responses* of “surrender,” “avoidance,” and “confrontation,” whereby avoidance is viewed in an active (“flight”/“divert attention”) or passive (“pacifying”/“self-soothing”) way. We therefore accessed the coping styles of participants during the pandemic and tested the hypotheses that participants exhibiting the behavior styles of “confrontation” and “diversion of attention” would significantly express low compliance, while participants exhibiting the styles of “surrender” and “self-soothing” would significantly express high compliance.

Our results indicate that the four factors established could be meaningfully termed “surrender,” “self-soothing,” “divert attention,” and “confrontation,” and that the four-factor categorization of coping styles is adoptable for schema-based therapy. Although here the term “divert attention” best reflects the factor established by the present items, it still corresponds with the concept of “active avoidance” or “escape” used in Young’s theory; as such, this grouping is principally in line with the fundamental theory of the coping styles in schema-oriented psychotherapy. Nevertheless, this observation may be useful for improvement of items in future research, as it may indicate a more complex underlying structure and may provide a step forward to resolve the ambiguity in conceptualization (*cf*. “freezing” by Atkinson, 2012; Faßbinder et al., 2016 versus Roediger and Zarbock, 2015) as mentioned above.

Further, our results show that the higher the coping style of “confrontation,” the less compliance can be expected, presenting a negative significant effect of “confrontation” on “compliance.” Our interpretation is that the coping style of “fighting against” in response to a threat is explained in psychology of motivation with the concept of “reactance” as the immediate response to restriction of freedom, particularly where the restrictions are not perceived as legitimate or justified and the restriction is not irrelevant (Graupmann et al., 2016). Furthermore, we did not find that either “self-soothing” or “divert attention” had a significant effect of on compliance. We consider that these passive and active forms of avoidance have no significant effect on compliance because they do not express proximity, which may be decisive for positive or negative compliance. While Karmakar et al. (2021) found self-soothing as a coping style during the present pandemic, Orgilés et al. (2021) found that avoidance-oriented styles were related to better psychological adaptation during the present pandemic. Further, our results show that the coping style of “surrender” correlates significantly with compliance, expressing a positive significant effect of “surrender” on “compliance.” This is not surprising, as the behavior style of succumbing-to or giving-in would imply abiding with sanctions. These findings seem in line with Blais et al. (2021), who found that “rule-followers” (*cf*. “CBS-surrender”) and “deliberate planners” (*cf*. CPN-efficacy) exhibit greater compliance in social distancing than those who are callous and antagonistic in personality.

### Concerns

Both traditions of schema-based therapy dwell in the context of personal distress and impairment of wellbeing, and within this study, they are conceptualized as worry or concerns in a non-clinical context. We therefore accessed participants’ concerns during the pandemic in our model and additionally tested the hypothesis that the stronger participants’ other existential concerns are, for example, with health issues, crime and social insecurity, setback at school or work, and financial or economic problems, the less compliant they are. This hypothesis could not be verified, showing that these existential issues do not show any statistically significant effect on whether individuals comply with anti-pandemic recommendations or not. Imbriano et al. (2021) likewise found no significant association of worry with compliance with health behaviors.

### Contribution to the Literature

Our research holds practical value to the literature input. First, to the best of our knowledge, this is the first study to investigate compliance behavior during a pandemic in light of the fundamentals of schema-based psychotherapy. Second, we believe that our findings can be beneficial for citizens, policymakers, risk managers, researchers, and experts in human behavior and health as our research contributes to the understanding of the psychological aspect of behavior during a pandemic. Microbiological and epidemiological data, although valuable, cannot exclusively inform pandemic policy; holistic approaches require a more in-depth knowledge of human behavior. Finally, our work presents a preliminary step toward reconciling the two independently developed traditions in schema-oriented psychotherapy.

### Limitations

It may be easy to endorse the finding that people tend to comply to anti-pandemic measures when they possess the coping style of submission. However, we venture to claim that compliance with pandemic measures does not necessarily signify subservience to authorities. Our findings do not demonstrate all motivations for compliance and non-compliance. There must be others: For instance, Dinić and Bodroža (2021) found that selfishness had negative effects on compliance with protective measures, and prosocial tendencies in general positively correlate with protective behaviors. Individuals may be non-compliant to demonstrate their disagreement with the authorities, or as an exhibition of power. It may even be an infantile act of defiance or influenced by peer pressure. Major barriers to compliance may include the complexity of the problem, the demands made by authorities and the steps to be taken, as well as misunderstanding the benefits of compliance. People could also fear side effects, be skeptical of costs, or feel suspicion backed by true experiences. Compliance may also be rooted in infantile servility, mental thralldom, or renunciation of responsibility. Further research is definitely needed.

There are some particular limitations we ought to note: Our study relied on self-reported responses, which are influenced by respondents’ imperfect memory or social desirability. Although data on personal needs and coping styles can primarily be self-reported, limitations inherent to self-report measures may affect results.

Further, it was convenient for participation to be online. However, persons with Internet access might not represent the general population. Thus, our findings are to be interpreted with respect to context and limitations, and generalized with care. Above, we illustrated the socio-demographic characteristics of participants and presented the descriptive statistics of the age of participants, not based on an idealized symmetric distribution (biological age grouping in 10 or 20 years) but grouped in the social-generational groups. Social generations are viewed as cohorts, whereby a cohort is seen as people within a delineated population who experienced significant range of same life event within a given historical time (Pilcher, 1994). Beyond the sociological dimension, the concept of a social generation provides a psychological dimension in the sense of belonging and shared identity to understand a socio-demographic (Biggs, 2007). Sandeen (2008), with reference to Strauss and Howe, views it as a “peer personality” and suggests that social-generational groups act as very meaningful segmentation in research. With 74% of participants belonging to Generation Z and Y, generalization to other social age groups has to be with care.

Though the scales of “self-esteem,” “confrontation,” “surrender,” and “diverted attention” are acceptable, slightly increasing the number of items would lead to a much better values for Cronbach’s alpha. If one would aim at standardizing the items for pandemic questionnaire based on schema therapy, it would be advantageous for items to be improved in later research. Our conclusions should therefore be taken as incentives for further exploration. Finally, our study was limited to a certain stage of the pandemic. Different results could be expected in a longitudinal study.

### Outlook

An extensive future study of the fundamental theories of schema-based psychotherapy during a pandemic may involve investigating whether clients’ problems during pandemic are schema-driven (e.g., “Vulnerability to Harm/Illness schema” or “Negativity/Pessimism schema”) or whether a schema that has been dormant in people can be activated by a pandemic (*cf*. Schema Therapy Bulletin 2020).

On investigating the theory of schema-oriented therapy during a pandemic, we focused on general, cross-cultural, and cross-gender trends although controlling for the effect of country, gender, education, and savings. Future studies may look at similar phenomena independently for different groups, such as gender, marital status, nationality, ethnicity, and education.

We believe science advances through benevolent criticism, counter-opinion, and suggestions for improvement. One would not generalize that all human behavior in all pandemics is exactly alike. Compliant behavior may also depend on other factors, for example, the kind or the novelty of the virus or its mutations, the policy details, and the duration of the pandemic. Similarly, compliance may depend on the individuals’ fears, sources of and level of critical analysis of information, and trust in authorities. Therefore, we contend that these additional aspects call for consideration.

Furthermore, developing a standardized questionnaire, particularly on the fulfillment of core psychological needs within the framework of schema-oriented psychotherapy, would be highly valuable for practitioners. We invite other researchers to this endeavor and believe that we have herewith provided a strong foundation.

## Practical Implications and Conclusion

A year has passed, and the world is still in the midst of the COVID-19 pandemic, which has led us to revisit one of psychology’s fundamental questions, *what determines human behavior*, and to examine it in the light of a contemporary and influential theory: schema-based psychotherapy.

Our findings present key insights that may (a) help effectively promote individual psychological consistency, (b) assist government’s regard of that, and (c) therefore, foster collective health-responsible behaviors.

First, these results teach that when drafting and communicating sanctions during a pandemic, the authorities should consider the driving force of behavior, i.e., the fulfillment of the CPNs. Authorities cannot limit their responsibility to promulgating restrictions that may impinge on fundamental human needs; to effectively control contagion during a pandemic, they must see the relevance of human need fulfillment. They should clearly highlight admissible and feasible actions that allow for the fulfillment of basic needs despite the context of a pandemic. Individuals are more prepared to comply when their topmost goals are taken into account and less prepared to comply when topmost goals are infringed.

Accordingly, the authorities should emphasize the superordinate goal of citizens’ efficacy and performance, and put power into citizen’s hands. Likewise, they should emphasize the superordinate goal of increasing pleasure and reducing pain.

Since restrictions can impair the fulfillment of the need for relationships amidst a pandemic, authorities should, for example, help build psychological supports for the population and provide and highlight alternative ways facilitate people finding their idiosyncratic *strategies* to meet, be acquainted with, relate to, and communicate, as well as develop and nourish relationships with others, in line with the anti-pandemic measures. For instance, to facilitate compliance among those who hold the CPN for *relationship* as a primary necessity during the pandemic, authorities need to emphasize that individuals may still socialize while sanitizing and limiting physical contact and that the pandemic restrictions ultimately aim at reinforcing possibilities for relationships in the long run and at reducing the chances of losing beloved ones.

Since restrictions also challenge self-determination, with regards to self-esteem, authorities should address individuals’ needs for honor, dignity, and autonomy, and make recommendations with due respect and without signs of defamation. Thus, in order to facilitate compliance among those who hold the CPN for *self-esteem* (dignity/autonomy), authorities must communicate with humility and respect, and authentically present themselves as being concerned about serving the people, without arrogance or demonstration of paternalism. They should also show gratitude for the valued contribution of compliance which the citizens eventually take autonomously.

Further, individuals have different ways of coping with challenges. While those with the coping style of surrender may easily comply with sanctions, particular attention should be given to individuals with the style of confrontation; their cooperation is not to be taken for granted; and reactance or the rejection of coerced blessings is to be expected. With due understanding of the concept of the behavior style of overcompensation/confrontation in schema therapy, there is need to make sure that reactance is not provoked, and when provoked that it is competently dealt with to the interest of the subject.

The key contribution of the current study is the importance of emphasizing individual’s superordinate goals (their basic needs) and behavior styles to support the imperative fulfillment of psychological needs, with respect to and for the CPNs that individuals are focused on at the particular time, rather than increasing compliance.

The key message is that supporting people from the perspective of the psychological needs which they are focused on fulfilling in the given moment and in respect of their behavior styles may be more affective for facilitating compliance than forcing or exhorting people to change/behave. Therefore, the effectiveness of anti-pandemic measures depends on the extent to which individual’s resources can be activated within their peculiarities.

A practical application of motivation *via* core psychological needs is illustrated in the concept called the “motive-oriented psychotherapeutic relationship” (Grawe, 2004; Caspar et al., 2005) in the tradition of Grawe. Accordingly, a decisive factor for effective therapy is the extent to which specific measures address the abilities within patient’s existing characteristics and subsequently activate their willingness to take action (Grawe and Grawe-Gerber, 1999). Dealing with reactance in therapy is illustrated in the concept of “empathic confrontation” in the tradition of Young and may be enrichened with concept of defense mechanisms in psychoanalysis. Defining precise mechanisms for political or medical authorities to achieve this during a pandemic is outside the purview of this article; for now, we allow them to grasp the importance of the concept.

We hope this research can assist in fostering understanding and cooperation between compliant and non-compliant people for the common goal of survival in any future pandemics. It must also be noted that we do not assert that compliance or adherence to authority is an ethical or moral value *per se* (*cf*. Milgram Experiment).^1^ Nevertheless, given the ongoing pandemic, and since compliance cannot be presumed, we believe that research that elucidates compliant behavior during a pandemic will aid in improving people’s lives during these trying times.

## Data Availability Statement

The original contributions presented in the study are included in the article/**Supplementary Material**, further inquiries can be directed to the corresponding author/s.

## Ethics Statement

The studies involving human participants were reviewed and approved by the Ethics Commission of the Institut für Verhaltenstherapie (Institute for Cognitive Behavior Psychotherapy Training and Research), AVM, Salzburg, Austria. The patients/participants provided their written informed consent to participate in this study.

## Author Contributions

CO conceptualized the study, designed the questionnaire, performed the primary data analysis, prepared the original draft of the manuscript, and contributed to the acquisition of funding. CO, ML, and BJ contributed to the improvement of theoretical and statistical model, analysis, and interpretation. All authors contributed to the manuscript revision, read, and approved the submitted version.

## Funding

The open access publication of this article has been made possible thanks to the funding of the Department of Psychology, the Faculty of Psychology and Sports Science, as well as the office of the Vice Rectorate for Research, all of the University of Innsbruck.

## Conflict of Interest

The authors declare that the research was conducted in the absence of any commercial or financial relationships that could be construed as a potential conflict of interest.

## Publisher’s Note

All claims expressed in this article are solely those of the authors and do not necessarily represent those of their affiliated organizations, or those of the publisher, the editors and the reviewers. Any product that may be evaluated in this article, or claim that may be made by its manufacturer, is not guaranteed or endorsed by the publisher.

## Footnotes

^1^Das Milgram Experiment (Video; 2021, April 12). YouTube. https://www.youtube.com/watch?v=0MzkVP2N9rw
