# Supplementary material for: Understanding Compliant Behavior During a Pandemic: Contribution From the Perspective of Schema-Based Psychotherapy
Source: Front Psychol. 2022 Feb 7;13:805987. doi: 10.3389/fpsyg.2022.805987 (PMC8859468; doi:10.3389/fpsyg.2022.805987)
Supplement: Supplementary file 1 [file Data_Sheet_1.pdf]

Supplementary Figures and Tables

**Supplementary Figure 1.**	Graphical representation of the research questions.
**Supplementary Figure 2.**	Scree plot of the exploratory factory analysis of compliance.
**Supplementary Figure 3.**	Scree plot of the exploratory factory analysis of core psychological needs.
**Supplementary Figure 4.**	Scree plot of the exploratory factory analysis of coping behavior styles.
**Supplementary Figure 5.**	Scree plot of the exploratory factory analysis of concerns.
**Supplementary Table 1.**	Research sample description table.
**Supplementary Table 2**	Exploratory factor analysis of compliance: communalities, and loadings.
**Supplementary Table 3**.	Exploratory factor analysis of core psychological needs: Rotated component matrix, communalities, and rotation sums of squared loadings.
**Supplementary Table 4.**	Exploratory factor analysis of behavior styles: Rotated component matrix, communalities, and rotation sums of squared loadings.
**Supplementary Table 5.**	Exploratory factor analysis of concerns: communalities, and loadings.

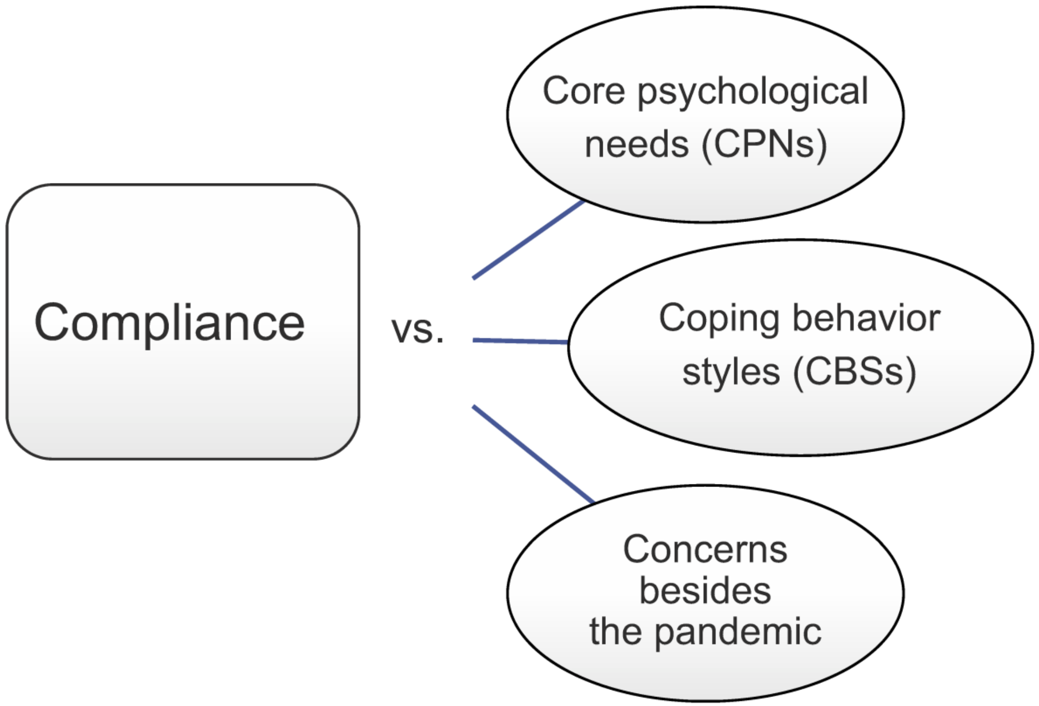

Supplementary Figure 1. Graphical representation of the research questions on whether compliance with anti-pandemic measures relates to core psychological needs, coping behavior styles, and concerns besides the pandemic.

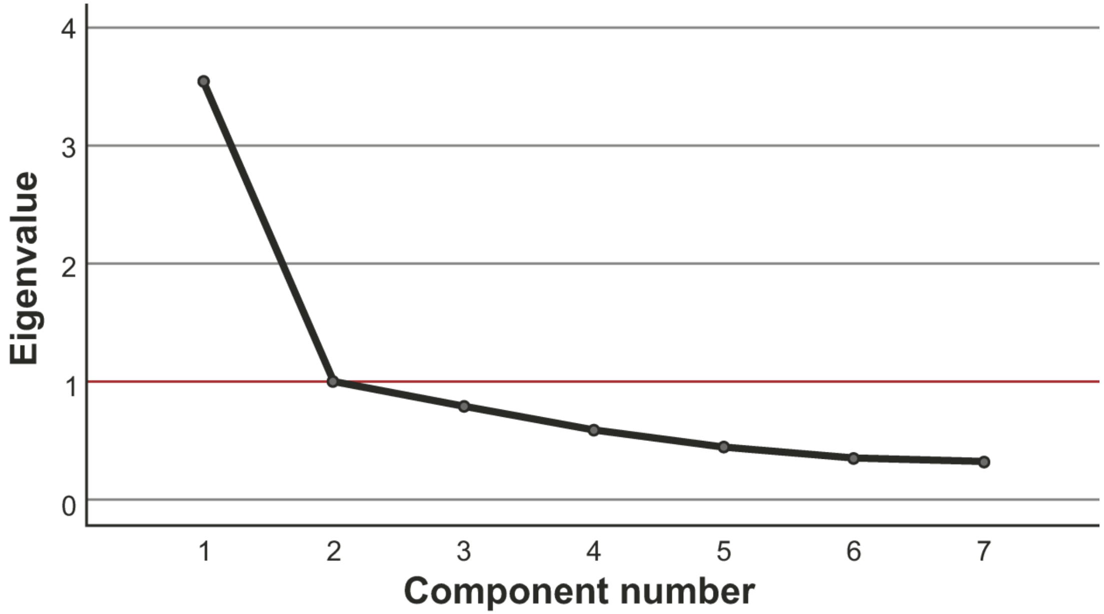

Supplementary Figure 2. Scree plot of the exploratory factory analysis of compliance showing that only one eigenvalue exceeds the Kaiser criterion of 1, thus confirming the unidimensionality of the compliance items.

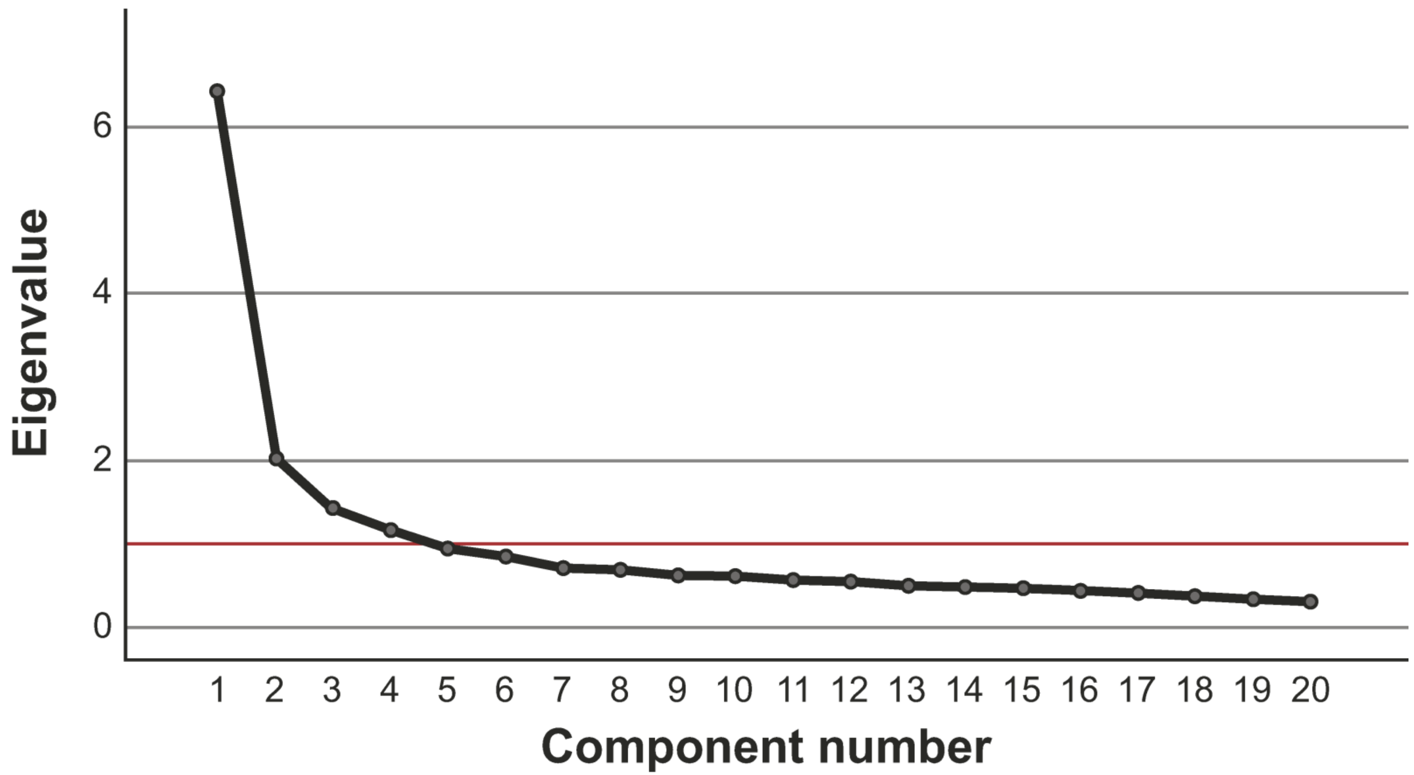

Supplementary Figure 3. Scree plot of the exploratory factory analysis of core psychological needs, with the eigenvalue of the factor above the Kaiser criterion of 1, denoting four as the optimal number of factors.

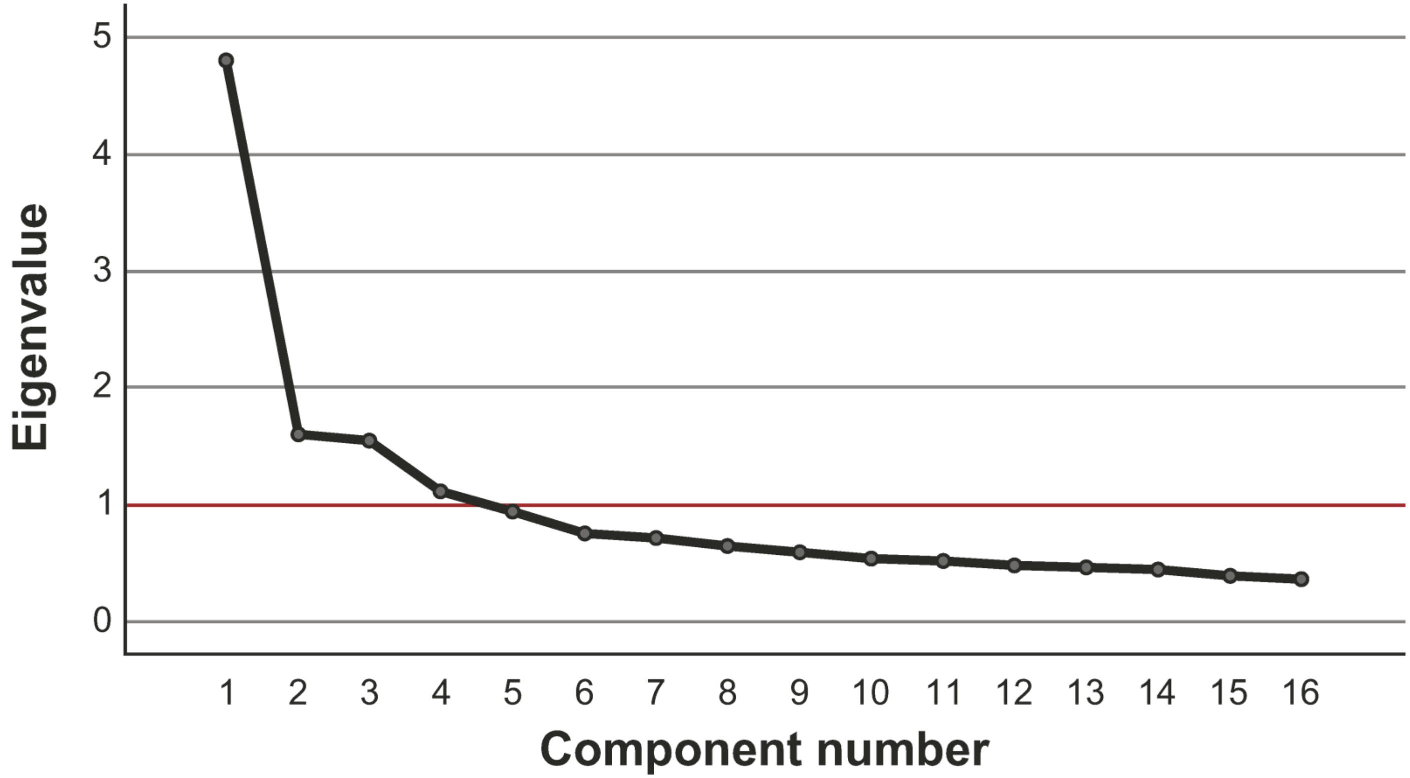

Supplementary Figure 4. Scree plot of the exploratory factory analysis of coping behavior styles with the eigenvalue of the factor above the Kaiser criterion of 1, denoting four as the optimal number of factors.

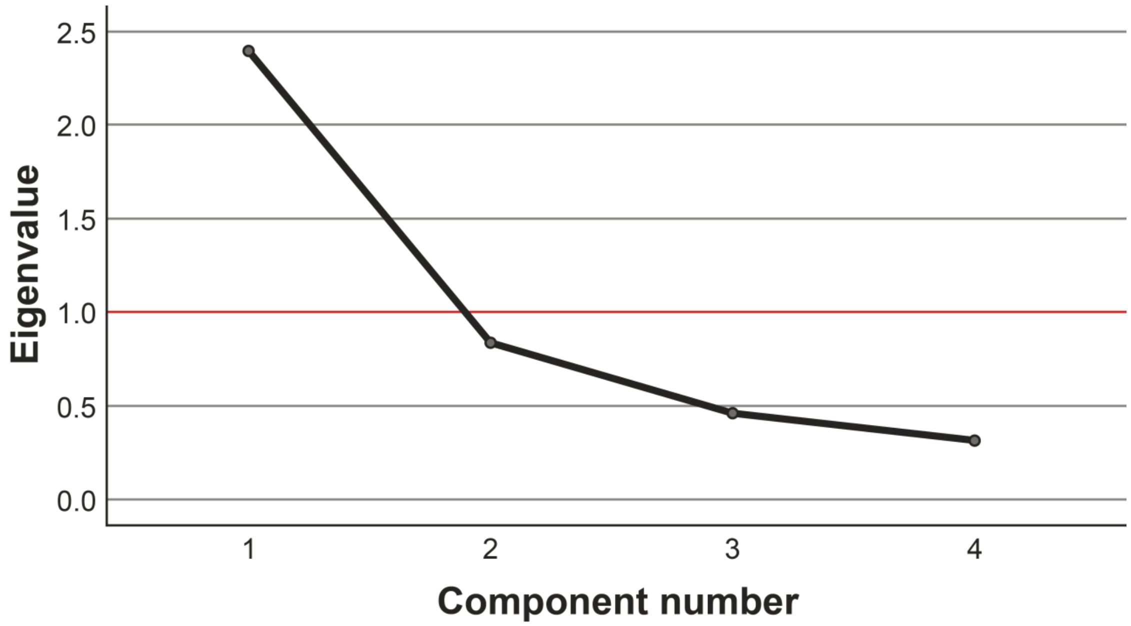

Supplementary Figure 5. Scree plot of the exploratory factory analysis of concerns showing that only one eigenvalue exceeds the Kaiser criterion of 1, thus confirming the unidimensionality of the concern items.

Supplementary Table 1. Research sample description table

		Frequency	Percent	Valid Percent	Cumulative Percent
N	Valid	740	740	740	740
	Missing	0	0	0	0
**Gender**	female	450	60.8	60.8	60.8
	male	290	39.2	39.2	100.0
**Country**	Others	26	3.5	3.5	3.5
	Austria	380	51.4	51.4	54.9
	Germany	44	5.9	5.9	60.8
	Nigeria	290	39.2	39.2	100.0
**Education**	Junior secondary education or lower	35	4.7	4.7	4.7
	Senior Secondary Education | High school diploma/A-levels	249	33.6	33.6	38.4
	Undergraduate degree / Diploma (OND, HND or equivalent)	121	16.4	16.4	54.7
	Bachelor degree (BA, BSc or equivalent)	175	23.6	23.6	78.4
	Master degree (MA, MSc, MPhil, or equivalent)	104	14.1	14.1	92.4
	Doctorate degree (PhD or equivalent)	23	3.1	3.1	95.5
	Other education, degree or qualification:	33	4.5	4.5	100.0
**Financial emergency reserves**	about 1 - 14 days	162	21.9	21.9	21.9
	about 1 - 3 months	241	32.6	32.6	54.5
	about 4 - 6 months	133	18.0	18.0	72.4
	longer than above mentioned	198	26.8	26.8	99.2
	otherwise:	6	0.8	0.8	100.0
**Age in five social groups**	Generation Z (Zoomer) 2009/12 - 1997 (8 - 23 years)	254	34.3	34.3	34.3
	Generation Y (Millennials) 1996 - 1981 (24 - 39 years)	294	39.7	39.7	74.1
	Generation X (Straddle?) 1980 - 1965 (40 - 55 years)	151	20.4	20.4	94.5
	Generation Baby Boomer & traditionalist 1964 - 1928 (56 - 92 years)	41	5.5	5.5	100.0

**Age**	Mean	Median	Mode	Std. Deviation	Variance	Minimum	Maximum
	32.06	28.50	18[a]	12.54	157.429	18	83

a. Multiple modes exist. The smallest value is shown.

Supplementary Table 2. Exploratory factor analysis of compliance: communalities and loadings.

Un-rotated Factor Matrix

	Factor	Communalities
	1	
cleanse my hands … or use hand-sanitizer more regularly	0.745	.556
wear a mask …to protect self and/or others	0.734	.539
avoid shaking hands or hugging people	0.812	.659
clean or disinfect surfaces I might touch more often	0.691	.477
have stopped or reduced travelling by public transport	0.664	.441
avoid group events or crowded places	0.818	.668
boost my immunity	0.455	.207
Rotation Sums of Squared Loadings	3.547	
% of Variance	50.667	
Cumulative %	50.667	

Extraction Method: Principal Component Analysis.

Supplementary Table 3. Exploratory factor analysis of core psychological needs (CPNs): Rotated component matrix, communalities, and rotation sums of squared loadings.

Rotated Component Matrix[a]

		Factors				Communalities
		1	2	3	4	
	Items	Efficacy	Pleasure	Relationship	Self-Esteem	
BV04_01	talk to others about the situation			0.665		0.562
BV04_02	prevent people from knowing how bad things are				0.724	0.527
BV04_03	improvise/develop a plan/strategy and follow it up	0.439			0.343	0.400
BV04_04	give myself a time-out (e.g., break, rest) …		0.718			0.582
BV04_05	look for something pleasant in what is happening		0.724			0.602
BV04_06	get sympathy from someone			0.687		0.531
BV04_07	try to still look good despite all adversities	0.447			0.477	0.476
BV04_08	try to get in control of the situation	0.680			0.308	0.570
BV04_09	re-energize myself (e.g., with exercise)	0.461	0.550			0.522
BV04_10	am inspired to do something creative	0.314	0.697			0.605
BV05_01	talk to someone I cherish about how I feel		0.312	0.644		0.594
BV05_02	try not to lose my reputation …	0.480			0.513	0.518
BV05_03	concentrate on coming up with possible solutions	0.772				0.684
BV05_04	establish/apply humor in order to ease the situation	0.553	0.316			0.456
BV05_05	learn something from it …	0.556	0.398			0.498
BV05_06	seek emotional support from friends or relatives			0.803		0.708
BV05_07	try not to be seen as the person with the worst case				0.725	0.562
BV05_08	double my efforts and try harder to handle the situation	0.483			0.616	0.627
BV05_09	make myself feel better by doing something pleasurable		0.604	0.318		0.584
BV05_10	seize the chance to develop closer relationships …		0.434	0.323	0.342	0.446
	Rotation Sums of Squared Loadings	3.120	2.935	2.534	2.467	
	% of Variance	15.600	14.675	12.668	12.335	
	Cumulative %	15.600	30.274	42.943	55.278	

Extraction Method: Principal Component Analysis. Rotation Method: Varimax with Kaiser Normalization. For better visualization only loadings >.30 are displayed.
a. Rotation converged in 7 iterations.

Supplementary Table 4. Exploratory factor analysis of coping behavior styles (CBSs): Rotated component matrix, communalities, and rotation sums of squared loadings.

Rotated Component Matrix[a]

		Factors				Communalities
		1	2	3	4	
	Items	Soothing	Confrontation	Surrender	Divert Attention	
BV06_01	accept what comes as my fate			0.786		0.647
BV06_02	engage in something different to escape from the strain …				0.789	0.667
BV06_03	refrain from doing anything until situation permits/problem declines	0.374		0.512		0.443
BV06_04	criticize or punish myself or others for the problem	0.682				0.574
BV06_06	take the next best option to what I wanted		0.567	0.380		0.563
~~BV06_07~~	run away/flee as far as I can	0.701				0.550
BV06_08	move on as if … there was no pandemic		0.727			0.607
BV06_09	stand my ground and fight … my way through		0.675			0.524
BV07_01	submit to circumstances as … destiny			0.797		0.690
BV07_02	turn to … other activities to take my mind off the problem				0.763	0.663
BV07_03	feel paralyzed and can't really do anything specific	0.761				0.606
BV07_04	combat/fight any problems tooth-and-nail		0.690			0.530
BV07_06	obey all instructions			0.454	0.395	0.380
BV07_07	daydream or develop fantasies … to forget the problem	0.496			0.479	0.494
BV07_08	try to calm myself by eating, drinking, gaming, etc.	0.721				0.552
~~BV07_09~~	confront/attack the cause of the problem	0.673	0.357			0.595
	Rotation Sums of Squared Loadings	3.073	2.148	2.011	1.852	
	% of Variance	19.207	13.427	12.571	11.575	
	Cumulative %	19.207	32.634	45.205	56.780	

Extraction Method: Principal Component Analysis. Rotation Method: Varimax with Kaiser Normalization. For better visualization only loadings >.30 are displayed.
a. Rotation converged in 7 iterations.

Supplementary Table 5. Exploratory factor analysis of concerns: communalities and loadings.

Un-rotated Factor Matrix

	Factor	Communalities
	1	
Concern: Health issues	0.588	.346
Concern: Crime and social insecurity	0.816	.666
Concern: Setback at school or work	0.826	.681
Concern: Financial or economic problem	0.834	.696
Rotation Sums of Squared Loadings	2.389	
% of Variance	59.731	
Cumulative %	59.731	

Extraction Method: Principal Component Analysis.
